# (*Z*)-Methyl 4-[3-(3-oxoquinuclidin-2-yl­idenemeth­yl)-1*H*-indol-1-ylmeth­yl]benzoate

**DOI:** 10.1107/S1600536808030018

**Published:** 2008-10-04

**Authors:** Thirupathi Reddy Yerram Reddy, Narsimha Reddy Penthala, Sean Parkin, Peter A. Crooks

**Affiliations:** a Department of Pharmaceutical Sciences, College of Pharmacy, University of Kentucky, Lexington, KY 40536, USA; b Department of Chemistry, University of Kentucky, Lexington, KY 40506, USA

## Abstract

The title compound, C_25_H_24_N_2_O_3_ was prepared by the reaction of (*Z*)-2-(1*H*-indol-3-ylmethyl­ene)-1-aza­bicyclo­[2.2.2]octan-3-one with methyl *p*-(bromo­meth­yl)benzoate, under phase-transfer catalytic (PTC) conditions using triethyl­benzyl­ammonium chloride and 50% *w*/*v* aqueous NaOH solution in dichloro­methane. The crystal structure indicates the presence of a double bond with *Z* geometry connecting the aza­bicyclic and indole groups.

## Related literature

For related structures, see: Mason *et al.* (2003 ▶); Zarza *et al.* (1988 ▶). For related bond angles, see: Wilson (1992 ▶).
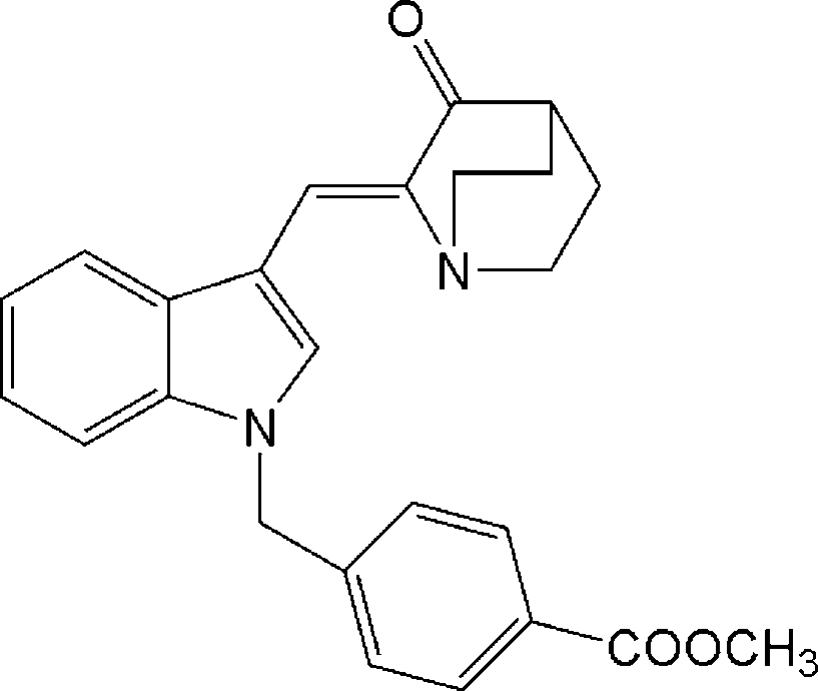


## Experimental

### 

#### Crystal data

C_25_H_24_N_2_O_3_
*M* *_r_* = 400.46
Triclinic, 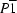

*a* = 9.8597 (3) Å
*b* = 10.3037 (3) Å
*c* = 11.3515 (4) Å
α = 106.6470 (14)°
β = 111.4372 (13)°
γ = 92.7863 (15)°
*V* = 1013.13 (6) Å^3^
*Z* = 2
Mo *K*α radiation
μ = 0.09 mm^−1^
*T* = 90.0 (2) K
0.55 × 0.50 × 0.25 mm

#### Data collection

Nonius KappaCCD diffractometer
Absorption correction: multi-scan (*SCALEPACK*; Otwinowski & Minor, 1997 ▶) *T*_min_ = 0.954, *T*_max_ = 0.979
11866 measured reflections
3974 independent reflections
2132 reflections with *I* > 2σ(*I*)
*R*_int_ = 0.068

#### Refinement

*R*[*F* ^2^ > 2σ(*F* ^2^)] = 0.054
*wR*(*F* ^2^) = 0.152
*S* = 0.97
3974 reflections
272 parameters
H-atom parameters constrained
Δρ_max_ = 0.27 e Å^−3^
Δρ_min_ = −0.25 e Å^−3^

### 

Data collection: *COLLECT* (Nonius, 1998 ▶); cell refinement: *SCALEPACK* (Otwinowski & Minor, 1997 ▶); data reduction: *DENZO-SMN* (Otwinowski & Minor, 1997 ▶); program(s) used to solve structure: *SHELXS97* (Sheldrick, 2008 ▶); program(s) used to refine structure: *SHELXL97* (Sheldrick, 2008 ▶); molecular graphics: *XP* in *SHELXTL* (Sheldrick, 2008 ▶); software used to prepare material for publication: *SHELX97* and local procedures.

## Supplementary Material

Crystal structure: contains datablocks global, I. DOI: 10.1107/S1600536808030018/om2262sup1.cif

Structure factors: contains datablocks I. DOI: 10.1107/S1600536808030018/om2262Isup2.hkl

Additional supplementary materials:  crystallographic information; 3D view; checkCIF report

## Figures and Tables

**Fig. 1.**
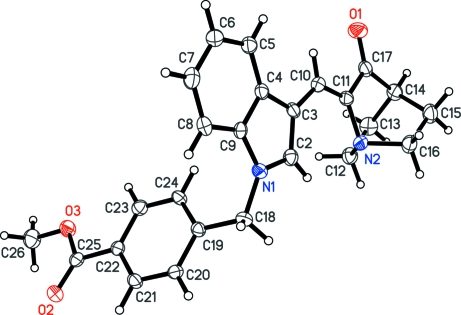
A view of the molecule with the atom numbering scheme. Displacement ellipsoids are drawn at the 50% probability level.

**Fig. 2.**
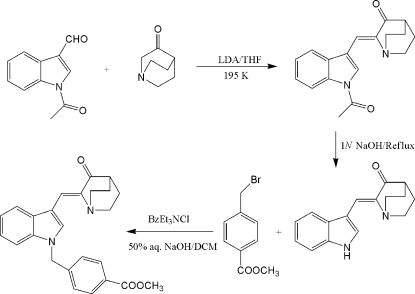
Reaction scheme.

## supplementary crystallographic information

### Comment

X-ray crystallography confirmed the molecular structure and the atom connectivity for the title compound, as illustrated in Fig. 1. The indole ring is planar with bond distances and angles comparable with those previously reported for other indole derivatives (Mason *et al.*, 2003; Zarza, *et al.*, 1988). The compound is the *Z* isomer, having the C11—C17 bond in a *trans* position with respect to the C3—C10 bond. The double bond (C10=C11) has a nearly planar atomic arrangement, since the r.m.s. deviation from the best plane passing through atoms N2, C11, C17, C10 and C3 is 0.0150 (14) Å. Deviations from ideal geometry are observed in the bond angles around atoms C3, C10 and C11. The C10=C11—C17 bond angle is close to the standard planar triangular value of 120°, whereas the C2=C3—C10, C3—C10=C11 and C10=C11—C17 bond angles are more distorted due to the strain induced by the C10=C11—C18=O1 conjugated double bond linkage. These bond angle deformations, which require little energy, are needed to release the intramolecular interactions between non-bonded atoms. In this molecule, the azabicyclic system presents very small distortions around atoms N2, C13, C14, C15, C16 and C17. The value of the C2—C3—C10—C11 torsion angle [-6.3 (4)°] indicates the deviation of the indole ring from the plane of the double bond connected to the azabicyclic ring. The C3—C10 bond length, when compared with the standard value for a single bond connecting a C_ar_ atom to a C*sp*^2^ atom (1.470 (15) Å; Wilson, 1992), suggests extensive conjugation, beginning at atom O1 and extending through to the indole ring. The bond angles in the azabicyclic system at C13, C14 and C15 are, on average, smaller than the standard tetrahedral value of 109.5°, while the bond angles at C12 and C16 are, on average, slightly larger than the ideal tetrahedral bond angle.

There are no significant intermolecular hydrogen-bonding interactions in the crystal structure. The packing is essentially stabilized *via* van der Waals forces.

### Experimental

To a stirred solution of diisopropylamine (1.923 g, 19 mmol) in THF (20 ml) at 273 K under nitrogen was added a solution of 2.0 *M* n-butyllithium (9 ml, 18.8 mmol) and the mixture stirred at 273 K for 30 min. To this solution at 273 K, was added 1-aza-bicyclo[2.2.2]octan-3-one hydrochloride (1.5 g, 9.28 mmol) in one portion and stirring continued until the mixture completely dissolved (20 min). The temperature was lowered to 195 K and a solution of 1-acetyl-1*H*-indole-3-carboxaldehyde (1.722 g, 9.2 mmol) in THF (25 ml) was added dropwise. Stirring was continued for 30 min at this temperature and then for 90 min at 273 K. The reaction mixture was poured into saturated NaHCO_3_ at 273 K and the resulting solution was extracted with CHCl_3_ (3 *x* 15 ml). The combined organic extracts were dried over anhydrous Na_2_SO_4_ and evaporated to afford (*Z*)-2-(1-acetyl- 1*H*-indol-3-ylmethylene)-1-azabicyclo[2.2.2]octan-3-one, which was subsequently refluxed with sodium hydroxide solution (25 ml, 1 N) for 30 min. The reaction mixture was cooled to room temperature, and the yellow solid that separated was collected by filtration, washed with cold water and dried to afford the (*Z*)-2-(1*H*-indol-3-ylmethylene) -1-azabicyclo[2.2.2]octan-3-one.

To a stirred mixture of (*Z*)-2-(1*H*-indol-3-ylmethylene) -1-azabicyclo[2.2.2]octan-3-one (1.0 g, 3.96 mmol), 50% *w*/*v* aqueous NaOH solution (1.52 g, 19 mmol) and benzyltriethylammonium chloride (0.172 g, 0.75 mmol) in dichloromethane (DCM, 25 ml) at room temperature was added methyl-*p*-(bromomethyl)benzoate (1.0 g, 4.0 mmol) in one portion, then the reaction mixture was stirred vigorously for 1 hr until no (*Z*)-2-(1*H*-indol-3-ylmethylene)-1-azabicyclo[2.2.2] octan-3-one was detected by TLC. The organic layer was separated, washed exhaustively with water, dried with Na_2_SO_4_ and evaporated to afford the crude product. Crystallization from methanol gave a yellow crystalline product of compound (I) that was suitable for X-ray analysis. ^1^H NMR (CDCl_3_): δ 1.98–2.00 (*m*, 4H), 2.61 (*p*, 1H), 2.93–3.02 (*m*, 2H), 3.08–3.15 (*m*, 2H), 3.88 (*s*, 3H), 5.42 (*s*, 2H), 7.14–7.22 (*m*, 5H), 7.47 (*s*, 1H), 7.87 (*d*, *J* = 7.2 Hz, 2H), 7.90 (*d*, *J* = 7.2 Hz, 1H), 8.38 (*s*, 1H); ^13^C NMR (CDCl_3_): δ 27.1, 41.1, 48.1, 51.0, 52.7, 110.6, 111.5, 118.2, 119.7, 121.6, 123.3, 126.9, 127.1, 129.3, 130.2, 130.6, 134.6, 136.4, 141.3, 142.2, 166.9, 205.6.

### Refinement

H atoms were found in difference Fourier maps and subsequently placed in idealized positions with constrained C—H distances of 0.98 Å (RCH_3_), 0.99 Å (*R*_2_CH_2_), 1.00 Å (*R*_3_CH) and 0.95 Å (C_Ar_H) with *U*_iso_(H) values set to either 1.5*U*_eq_ (RCH_3_ only) or 1.2*U*_eq_ of the attached C atom.

### Crystal data

**Table:**

C_25_H_24_N_2_O_3_	*Z* = 2
*M**_r_* = 400.46	*F*(000) = 424
Triclinic, *P*1	*D*_x_ = 1.313 Mg m^−^^3^
Hall symbol: -P 1	Mo *K*α radiation, λ = 0.71073 Å
*a* = 9.8597 (3) Å	Cell parameters from 3599 reflections
*b* = 10.3037 (3) Å	θ = 1.0–27.5°
*c* = 11.3515 (4) Å	µ = 0.09 mm^−^^1^
α = 106.6470 (14)°	*T* = 90 K
β = 111.4372 (13)°	Irregular block, colourless
γ = 92.7863 (15)°	0.55 × 0.50 × 0.25 mm
*V* = 1013.13 (6) Å^3^	

### Data collection

**Table:**

Nonius KappaCCD diffractometer	3974 independent reflections
Radiation source: fine-focus sealed tube	2132 reflections with *I* > 2σ(*I*)
graphite	*R*_int_ = 0.068
Detector resolution: 18 pixels mm^-1^	θ_max_ = 26.0°, θ_min_ = 2.0°
ω scans at fixed χ = 55°	*h* = −12→11
Absorption correction: multi-scan (SCALEPACK; Otwinowski & Minor, 1997)	*k* = −12→12
*T*_min_ = 0.954, *T*_max_ = 0.979	*l* = −13→13
11866 measured reflections	

### Refinement

**Table:**

Refinement on *F*^2^	Primary atom site location: structure-invariant direct methods
Least-squares matrix: full	Secondary atom site location: difference Fourier map
*R*[*F*^2^ > 2σ(*F*^2^)] = 0.054	Hydrogen site location: inferred from neighbouring sites
*wR*(*F*^2^) = 0.152	H-atom parameters constrained
*S* = 0.97	*w* = 1/[σ^2^(*F*_o_^2^) + (0.0735*P*)^2^] where *P* = (*F*_o_^2^ + 2*F*_c_^2^)/3
3974 reflections	(Δ/σ)_max_ = 0.002
272 parameters	Δρ_max_ = 0.27 e Å^−^^3^
0 restraints	Δρ_min_ = −0.25 e Å^−^^3^

### Special details

**Table:**

Geometry. All e.s.d.'s (except the e.s.d. in the dihedral angle between two l.s. planes) are estimated using the full covariance matrix. The cell e.s.d.'s are taken into account individually in the estimation of e.s.d.'s in distances, angles and torsion angles; correlations between e.s.d.'s in cell parameters are only used when they are defined by crystal symmetry. An approximate (isotropic) treatment of cell e.s.d.'s is used for estimating e.s.d.'s involving l.s. planes.
Refinement. Refinement of *F*^2^ against ALL reflections. The weighted *R*-factor *wR* and goodness of fit *S* are based on *F*^2^, conventional *R*-factors *R* are based on *F*, with *F* set to zero for negative *F*^2^. The threshold expression of *F*^2^ > 2σ(*F*^2^) is used only for calculating *R*-factors(gt) *etc*. and is not relevant to the choice of reflections for refinement. *R*-factors based on *F*^2^ are statistically about twice as large as those based on *F*, and *R*- factors based on ALL data will be even larger.

### Fractional atomic coordinates and isotropic or equivalent isotropic displacement parameters (Å^2^)

**Table:**

	*x*	*y*	*z*	*U*_iso_*/*U*_eq_	
N1	0.4526 (2)	0.7860 (2)	0.42974 (19)	0.0196 (5)	
N2	0.7265 (2)	0.6780 (2)	0.19469 (19)	0.0217 (5)	
O1	0.58592 (19)	0.79547 (19)	−0.09183 (17)	0.0349 (5)	
O2	−0.05653 (18)	0.23941 (17)	0.44169 (17)	0.0270 (5)	
O3	−0.11508 (18)	0.24052 (17)	0.23058 (16)	0.0274 (5)	
C2	0.5260 (3)	0.7563 (2)	0.3468 (2)	0.0206 (6)	
H2	0.5975	0.6972	0.3523	0.025*	
C3	0.4822 (3)	0.8236 (2)	0.2539 (2)	0.0204 (6)	
C4	0.3734 (3)	0.9016 (2)	0.2827 (2)	0.0197 (6)	
C5	0.2910 (3)	0.9933 (3)	0.2293 (2)	0.0229 (6)	
H5	0.3002	1.0142	0.1560	0.027*	
C6	0.1965 (3)	1.0527 (3)	0.2845 (3)	0.0254 (6)	
H6	0.1414	1.1159	0.2495	0.031*	
C7	0.1802 (3)	1.0215 (3)	0.3912 (3)	0.0277 (7)	
H7	0.1130	1.0629	0.4263	0.033*	
C8	0.2591 (3)	0.9322 (2)	0.4463 (2)	0.0222 (6)	
H8	0.2475	0.9102	0.5182	0.027*	
C9	0.3569 (3)	0.8754 (2)	0.3919 (2)	0.0194 (6)	
C10	0.5266 (3)	0.8148 (3)	0.1450 (2)	0.0214 (6)	
H10	0.4756	0.8624	0.0864	0.026*	
C11	0.6295 (3)	0.7491 (3)	0.1140 (2)	0.0208 (6)	
C12	0.7098 (3)	0.5330 (3)	0.1114 (3)	0.0268 (7)	
H12A	0.6084	0.4851	0.0846	0.032*	
H12B	0.7800	0.4860	0.1652	0.032*	
C13	0.7389 (3)	0.5234 (3)	−0.0153 (3)	0.0319 (7)	
H13A	0.8200	0.4708	−0.0171	0.038*	
H13B	0.6490	0.4752	−0.0964	0.038*	
C14	0.7819 (3)	0.6700 (3)	−0.0135 (2)	0.0256 (6)	
H14	0.8014	0.6682	−0.0944	0.031*	
C15	0.9188 (3)	0.7419 (3)	0.1160 (3)	0.0314 (7)	
H15A	0.9490	0.8368	0.1202	0.038*	
H15B	1.0020	0.6915	0.1173	0.038*	
C16	0.8814 (3)	0.7456 (3)	0.2379 (3)	0.0272 (7)	
H16A	0.9500	0.6982	0.2926	0.033*	
H16B	0.8952	0.8424	0.2945	0.033*	
C17	0.6562 (3)	0.7455 (3)	−0.0068 (2)	0.0236 (6)	
C18	0.4596 (3)	0.7224 (3)	0.5299 (2)	0.0222 (6)	
H18A	0.4622	0.7937	0.6110	0.027*	
H18B	0.5527	0.6849	0.5552	0.027*	
C19	0.3302 (3)	0.6077 (2)	0.4828 (2)	0.0184 (6)	
C20	0.3106 (3)	0.5504 (3)	0.5739 (2)	0.0206 (6)	
H20	0.3792	0.5828	0.6646	0.025*	
C21	0.1933 (3)	0.4476 (2)	0.5343 (2)	0.0214 (6)	
H21	0.1813	0.4099	0.5978	0.026*	
C22	0.0920 (3)	0.3983 (2)	0.4013 (2)	0.0183 (6)	
C23	0.1106 (3)	0.4535 (3)	0.3092 (2)	0.0221 (6)	
H23	0.0423	0.4200	0.2183	0.027*	
C24	0.2288 (3)	0.5577 (2)	0.3496 (2)	0.0207 (6)	
H24	0.2409	0.5953	0.2861	0.025*	
C25	−0.0330 (3)	0.2863 (2)	0.3636 (2)	0.0208 (6)	
C26	−0.2337 (3)	0.1258 (3)	0.1858 (3)	0.0345 (7)	
H26A	−0.1959	0.0566	0.2275	0.052*	
H26B	−0.2716	0.0847	0.0882	0.052*	
H26C	−0.3137	0.1587	0.2117	0.052*	

### Atomic displacement parameters (Å^2^)

**Table:**

	*U*^11^	*U*^22^	*U*^33^	*U*^12^	*U*^13^	*U*^23^
N1	0.0217 (12)	0.0210 (12)	0.0198 (11)	0.0051 (10)	0.0104 (10)	0.0090 (10)
N2	0.0193 (12)	0.0247 (12)	0.0204 (11)	0.0035 (10)	0.0062 (10)	0.0086 (10)
O1	0.0373 (12)	0.0493 (13)	0.0296 (11)	0.0136 (10)	0.0170 (10)	0.0235 (10)
O2	0.0263 (10)	0.0302 (11)	0.0287 (11)	0.0038 (9)	0.0131 (9)	0.0131 (9)
O3	0.0243 (10)	0.0293 (11)	0.0234 (10)	−0.0036 (9)	0.0053 (9)	0.0076 (8)
C2	0.0196 (14)	0.0180 (14)	0.0250 (15)	0.0046 (12)	0.0105 (12)	0.0059 (12)
C3	0.0188 (14)	0.0230 (14)	0.0203 (14)	−0.0009 (12)	0.0079 (12)	0.0092 (12)
C4	0.0174 (13)	0.0189 (14)	0.0209 (14)	−0.0009 (12)	0.0064 (12)	0.0058 (12)
C5	0.0209 (14)	0.0250 (15)	0.0214 (14)	0.0007 (13)	0.0072 (12)	0.0078 (12)
C6	0.0239 (15)	0.0237 (14)	0.0306 (15)	0.0050 (13)	0.0109 (13)	0.0113 (13)
C7	0.0215 (15)	0.0276 (15)	0.0343 (16)	0.0041 (13)	0.0138 (13)	0.0068 (13)
C8	0.0211 (14)	0.0208 (14)	0.0241 (15)	−0.0015 (12)	0.0109 (13)	0.0046 (12)
C9	0.0147 (13)	0.0180 (14)	0.0199 (14)	−0.0027 (12)	0.0030 (12)	0.0040 (11)
C10	0.0185 (14)	0.0221 (14)	0.0221 (14)	0.0020 (12)	0.0059 (12)	0.0080 (12)
C11	0.0200 (14)	0.0245 (14)	0.0176 (13)	0.0011 (12)	0.0067 (12)	0.0077 (12)
C12	0.0257 (15)	0.0233 (15)	0.0303 (15)	0.0039 (13)	0.0112 (13)	0.0071 (13)
C13	0.0362 (17)	0.0305 (16)	0.0279 (15)	0.0064 (14)	0.0147 (14)	0.0050 (13)
C14	0.0269 (15)	0.0337 (16)	0.0207 (14)	0.0060 (14)	0.0145 (13)	0.0085 (13)
C15	0.0232 (15)	0.0402 (17)	0.0320 (16)	0.0006 (14)	0.0134 (13)	0.0111 (14)
C16	0.0216 (15)	0.0319 (16)	0.0267 (15)	0.0032 (13)	0.0092 (13)	0.0083 (13)
C17	0.0217 (14)	0.0268 (15)	0.0215 (14)	−0.0020 (13)	0.0080 (12)	0.0083 (12)
C18	0.0246 (14)	0.0255 (14)	0.0198 (14)	0.0056 (12)	0.0093 (12)	0.0112 (12)
C19	0.0200 (14)	0.0180 (13)	0.0208 (14)	0.0053 (12)	0.0110 (12)	0.0072 (12)
C20	0.0198 (14)	0.0256 (15)	0.0201 (14)	0.0081 (12)	0.0086 (12)	0.0113 (12)
C21	0.0235 (14)	0.0239 (14)	0.0242 (15)	0.0076 (13)	0.0143 (12)	0.0118 (12)
C22	0.0186 (14)	0.0187 (13)	0.0213 (14)	0.0079 (12)	0.0108 (12)	0.0073 (12)
C23	0.0226 (14)	0.0232 (14)	0.0193 (14)	0.0051 (12)	0.0078 (12)	0.0056 (12)
C24	0.0234 (15)	0.0241 (14)	0.0196 (14)	0.0041 (13)	0.0118 (12)	0.0100 (12)
C25	0.0212 (15)	0.0215 (14)	0.0246 (15)	0.0103 (12)	0.0116 (13)	0.0101 (13)
C26	0.0282 (16)	0.0317 (16)	0.0336 (17)	−0.0053 (14)	0.0063 (14)	0.0058 (14)

### Geometric parameters (Å, °)

**Table:**

N1—C2	1.364 (3)	C12—H12B	0.9900
N1—C9	1.392 (3)	C13—C14	1.541 (3)
N1—C18	1.449 (3)	C13—H13A	0.9900
N2—C11	1.450 (3)	C13—H13B	0.9900
N2—C12	1.486 (3)	C14—C17	1.508 (3)
N2—C16	1.487 (3)	C14—C15	1.536 (3)
O1—C17	1.228 (3)	C14—H14	1.0000
O2—C25	1.208 (3)	C15—C16	1.549 (3)
O3—C25	1.349 (3)	C15—H15A	0.9900
O3—C26	1.453 (3)	C15—H15B	0.9900
C2—C3	1.377 (3)	C16—H16A	0.9900
C2—H2	0.9500	C16—H16B	0.9900
C3—C10	1.435 (3)	C18—C19	1.517 (3)
C3—C4	1.447 (3)	C18—H18A	0.9900
C4—C5	1.401 (3)	C18—H18B	0.9900
C4—C9	1.404 (3)	C19—C20	1.392 (3)
C5—C6	1.378 (3)	C19—C24	1.397 (3)
C5—H5	0.9500	C20—C21	1.375 (3)
C6—C7	1.398 (3)	C20—H20	0.9500
C6—H6	0.9500	C21—C22	1.394 (3)
C7—C8	1.374 (3)	C21—H21	0.9500
C7—H7	0.9500	C22—C23	1.386 (3)
C8—C9	1.392 (3)	C22—C25	1.492 (3)
C8—H8	0.9500	C23—C24	1.389 (3)
C10—C11	1.343 (3)	C23—H23	0.9500
C10—H10	0.9500	C24—H24	0.9500
C11—C17	1.478 (3)	C26—H26A	0.9800
C12—C13	1.544 (3)	C26—H26B	0.9800
C12—H12A	0.9900	C26—H26C	0.9800
			
C2—N1—C9	108.62 (19)	C17—C14—H14	111.3
C2—N1—C18	125.6 (2)	C15—C14—H14	111.3
C9—N1—C18	125.4 (2)	C13—C14—H14	111.3
C11—N2—C12	109.37 (18)	C14—C15—C16	109.1 (2)
C11—N2—C16	107.79 (19)	C14—C15—H15A	109.9
C12—N2—C16	108.0 (2)	C16—C15—H15A	109.9
C25—O3—C26	114.8 (2)	C14—C15—H15B	109.9
N1—C2—C3	110.7 (2)	C16—C15—H15B	109.9
N1—C2—H2	124.7	H15A—C15—H15B	108.3
C3—C2—H2	124.7	N2—C16—C15	111.3 (2)
C2—C3—C10	128.3 (2)	N2—C16—H16A	109.4
C2—C3—C4	105.9 (2)	C15—C16—H16A	109.4
C10—C3—C4	125.7 (2)	N2—C16—H16B	109.4
C5—C4—C9	118.2 (2)	C15—C16—H16B	109.4
C5—C4—C3	134.6 (2)	H16A—C16—H16B	108.0
C9—C4—C3	107.2 (2)	O1—C17—C11	125.4 (2)
C6—C5—C4	119.1 (2)	O1—C17—C14	123.8 (2)
C6—C5—H5	120.4	C11—C17—C14	110.8 (2)
C4—C5—H5	120.4	N1—C18—C19	113.29 (19)
C5—C6—C7	121.2 (2)	N1—C18—H18A	108.9
C5—C6—H6	119.4	C19—C18—H18A	108.9
C7—C6—H6	119.4	N1—C18—H18B	108.9
C8—C7—C6	121.4 (2)	C19—C18—H18B	108.9
C8—C7—H7	119.3	H18A—C18—H18B	107.7
C6—C7—H7	119.3	C20—C19—C24	118.6 (2)
C7—C8—C9	116.9 (2)	C20—C19—C18	119.7 (2)
C7—C8—H8	121.5	C24—C19—C18	121.6 (2)
C9—C8—H8	121.5	C21—C20—C19	121.0 (2)
N1—C9—C8	129.2 (2)	C21—C20—H20	119.5
N1—C9—C4	107.7 (2)	C19—C20—H20	119.5
C8—C9—C4	123.2 (2)	C20—C21—C22	120.3 (2)
C11—C10—C3	129.5 (2)	C20—C21—H21	119.9
C11—C10—H10	115.3	C22—C21—H21	119.9
C3—C10—H10	115.3	C23—C22—C21	119.5 (2)
C10—C11—N2	124.3 (2)	C23—C22—C25	122.4 (2)
C10—C11—C17	122.2 (2)	C21—C22—C25	118.1 (2)
N2—C11—C17	113.4 (2)	C22—C23—C24	120.1 (2)
N2—C12—C13	111.7 (2)	C22—C23—H23	120.0
N2—C12—H12A	109.3	C24—C23—H23	120.0
C13—C12—H12A	109.3	C23—C24—C19	120.6 (2)
N2—C12—H12B	109.3	C23—C24—H24	119.7
C13—C12—H12B	109.3	C19—C24—H24	119.7
H12A—C12—H12B	107.9	O2—C25—O3	123.6 (2)
C14—C13—C12	108.8 (2)	O2—C25—C22	124.4 (2)
C14—C13—H13A	109.9	O3—C25—C22	111.9 (2)
C12—C13—H13A	109.9	O3—C26—H26A	109.5
C14—C13—H13B	109.9	O3—C26—H26B	109.5
C12—C13—H13B	109.9	H26A—C26—H26B	109.5
H13A—C13—H13B	108.3	O3—C26—H26C	109.5
C17—C14—C15	107.8 (2)	H26A—C26—H26C	109.5
C17—C14—C13	107.2 (2)	H26B—C26—H26C	109.5
C15—C14—C13	107.9 (2)		
			
C9—N1—C2—C3	0.0 (3)	C12—C13—C14—C15	57.9 (3)
C18—N1—C2—C3	173.2 (2)	C17—C14—C15—C16	56.2 (3)
N1—C2—C3—C10	−176.5 (2)	C13—C14—C15—C16	−59.3 (3)
N1—C2—C3—C4	0.3 (3)	C11—N2—C16—C15	−58.5 (3)
C2—C3—C4—C5	177.7 (3)	C12—N2—C16—C15	59.6 (3)
C10—C3—C4—C5	−5.4 (4)	C14—C15—C16—N2	0.6 (3)
C2—C3—C4—C9	−0.5 (3)	C10—C11—C17—O1	−2.9 (4)
C10—C3—C4—C9	176.4 (2)	N2—C11—C17—O1	178.2 (2)
C9—C4—C5—C6	−0.7 (3)	C10—C11—C17—C14	177.6 (2)
C3—C4—C5—C6	−178.7 (3)	N2—C11—C17—C14	−1.3 (3)
C4—C5—C6—C7	−0.9 (4)	C15—C14—C17—O1	123.5 (3)
C5—C6—C7—C8	1.0 (4)	C13—C14—C17—O1	−120.5 (3)
C6—C7—C8—C9	0.6 (4)	C15—C14—C17—C11	−57.0 (3)
C2—N1—C9—C8	179.3 (2)	C13—C14—C17—C11	58.9 (3)
C18—N1—C9—C8	6.0 (4)	C2—N1—C18—C19	−98.3 (3)
C2—N1—C9—C4	−0.4 (3)	C9—N1—C18—C19	73.8 (3)
C18—N1—C9—C4	−173.6 (2)	N1—C18—C19—C20	−170.9 (2)
C7—C8—C9—N1	178.2 (2)	N1—C18—C19—C24	8.9 (3)
C7—C8—C9—C4	−2.3 (3)	C24—C19—C20—C21	−0.6 (4)
C5—C4—C9—N1	−178.0 (2)	C18—C19—C20—C21	179.2 (2)
C3—C4—C9—N1	0.5 (3)	C19—C20—C21—C22	0.3 (4)
C5—C4—C9—C8	2.3 (3)	C20—C21—C22—C23	0.2 (3)
C3—C4—C9—C8	−179.1 (2)	C20—C21—C22—C25	179.8 (2)
C2—C3—C10—C11	−6.3 (4)	C21—C22—C23—C24	−0.4 (3)
C4—C3—C10—C11	177.4 (2)	C25—C22—C23—C24	180.0 (2)
C3—C10—C11—N2	−4.2 (4)	C22—C23—C24—C19	0.1 (4)
C3—C10—C11—C17	177.0 (2)	C20—C19—C24—C23	0.4 (3)
C12—N2—C11—C10	123.9 (3)	C18—C19—C24—C23	−179.4 (2)
C16—N2—C11—C10	−118.9 (3)	C26—O3—C25—O2	−1.8 (3)
C12—N2—C11—C17	−57.2 (3)	C26—O3—C25—C22	176.60 (19)
C16—N2—C11—C17	60.0 (3)	C23—C22—C25—O2	−176.8 (2)
C11—N2—C12—C13	55.9 (3)	C21—C22—C25—O2	3.6 (4)
C16—N2—C12—C13	−61.2 (3)	C23—C22—C25—O3	4.8 (3)
N2—C12—C13—C14	1.8 (3)	C21—C22—C25—O3	−174.8 (2)
C12—C13—C14—C17	−58.0 (3)		
